# Efficiency of biochar, nitrogen addition, and microbial agent amendments in remediation of soil properties and microbial community in Qilian Mountains mine soils

**DOI:** 10.1002/ece3.7715

**Published:** 2021-06-19

**Authors:** Junqia Kong, Zhibin He, Longfei Chen, Rong Yang, Jun Du

**Affiliations:** ^1^ Linze Inland River Basin Research Station Chinese Ecosystem Research Network Key Laboratory of Eco‐hydrology of Inland River Basin Northwest Institute of Eco‐Environment and Resources Chinese Academy of Sciences Lanzhou China; ^2^ University of Chinese Academy of Sciences Beijing China

**Keywords:** biochar, C/N ratio, microbial agent, microbial community, mine soil, N fertilizer, soil quality

## Abstract

Lacking systematic evaluations in soil quality and microbial community recovery after different amendments addition limits optimization of amendments combination in coal mine soils. We performed a short‐term incubation experiment with a varying temperature over 12 weeks to assess the effects of three amendments (biochar: C; nitrogen fertilizer at three levels: N‐N1~N3; microbial agent at two levels: M‐M1~M2) based on C/N ratio (regulated by biochar and N level: 35:1, 25:1, 12.5:1) on mine soil properties and microbial community in the Qilian Mountains, China. Over the incubation period, soil pH and MBC/MBN were significantly lower than unamended treatment in N addition and C + M + N treatments, respectively. Soil organic carbon (SOC), total nitrogen (TN), available nitrogen (AN), available phosphorus (AP), available potassium (AK), microbial biomass carbon (MBC), and nitrogen (MBN) contents increased significantly in all amended treatments (*p* < .001). Higher AP, AK, MBC, MBN, and lower MBC/MBN were observed in N2‐treated soil (corresponding to C/N ratio of 25:1). Meanwhile, N2‐treated soil significantly increased species richness and diversity of soil bacterial community (*p* < .05). Principal coordinate analysis further showed that soil bacterial community compositions were significantly separated by N level. C‐M‐N treatments significantly increased the relative abundance (>1%) of the bacterial phyla Bacteroidetes and Firmicutes, and decreased the relative abundance of fungal phyla Chytridiomycota (*p* < .05). Redundancy analysis illustrated the importance of soil nutrients in explaining variability in bacterial community composition (74.73%) than fungal composition (35.0%). Our results indicated that N addition based on biochar and M can improve soil quality by neutralizing soil pH and increasing soil nutrient contents in short‐term, and the appropriate C/N ratio (25:1) can better promote microbial mass, richness, and diversity of soil bacterial community. Our study provided a new insight for achieving restoration of damaged habitats by changing microbial structure, diversity, and mass by regulating C/N ratio of amendments.

## Graphical abstract

The first attempts to combine three amendments to restore mine soils. N2‐treated soil can promote bacterial species richness and diversity restoration. A new insight for mine soil restoration by regulating C/N ratio by amendments

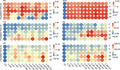

## 1 INTRODUCTION

Mining activities in mountainous areas alter soil properties, nutrient availability, and microbial activity, posing environmental threats associated with land degradation, water and soil erosion, and loss of biodiversity (Ahirwal & Maiti, 2018; Garbin et al., 2018; Józefowska et al., 2017). Effective soil reclamation processes become urgent and arduous tasks aimed at recovery of the destroyed environment to a self‐sustaining state in opencast mining areas. An environmentally sustainable method for achieving soil reclamation in mining areas is the use of soil amendments (Asensio et al., 2013; Zornoza et al., 2013).

The success of amendments in soil reclamation can be evaluated mainly on two aspects: efficient increase in soil nutrients to support vegetation demand, and contribute to growth of soil microbial community (Chen, He, et al., 2020; Chen, Mo, et al., 2020; Zornoza et al., 2016). However, applications of various amendments lack systematic evaluations of their effectiveness in restoring mining ecosystems, limiting the selection of materials and amendments for soil reclamation and constraining critical improvements in soil quality and the growth of soil microbial biomass in mining areas.

Biochar amendments have been recently widely and successfully used in mine soil reclamation (Lehmann et al., 2011; Moreno‐Barriga et al., 2017). Previous studies have reported the positive effects on soil quality and health of biochar created through the pyrolysis of organic residues (Lehmann et al., 2011; Marchetti et al., 2012). The addition of biochar to mine soils can efficiently contribute to the formation of soil organic matter, retention of nutrients, and sequestration of heavy metals; meanwhile, biochar additions can alter some soil microbial communities composition (Grossman et al., 2010) and stimulate the growth of soil microbial communities (Li et al., 2018; Moreno‐Barriga et al., 2017). These benefits of biochar indicate that biochar can be used in combination with other amendments to enhance positive effects on mine soils. Soil microorganisms play key roles in ecological functioning of ecosystems, including regulating soil organic matter decomposition and carbon stabilization, and mediating nutrient cycling (Pan et al., 2018; Sun et al., 2016). However, extreme soil conditions caused by severe mining disturbance usually have a negative influence on the recovery of soil microbial community diversity and mass (de Quadros et al., 2016). Previous studies of reclaimed mine soils indicated that microbial biomass and diversity may take 5 to 14 years or longer to recover to undisturbed soil levels (Dangi et al., 2012). Thus, given the importance of soil microbial community to damaged mining habitat, we try to add microbial agents on the basis of biochar amendments in order to activate microbial activity. It is important to verify whether the addition of microbial agents combined with biochar can activate soil microbial activity and may give new insights on how to promote soil microbial recovery in damaged habitats.

Biochar additions to soil can also absorb mineral nitrogen, which can reduce nitrate–nitrogen (NO_3_^−^–N) leaching, increase ammonium–nitrogen (NH_4_^+^‐N) retention, and improve the use efficiency of nitrogen fertilizer (Ameloot et al., 2015; Clough et al., 2013). Studies have shown that the combined application of biochar and nitrogen fertilizer had significant effects on soil nutrient contents, microbial biomass carbon, nitrogen, and crop yields in agricultural lands (Zheng et al., 2012; Zhu et al., 2014). However, there are few reports on the combined application of biochar and nitrogen fertilizer in mine soils. In mine soils, the effects of amendments on soil physicochemical properties, microbial biomass, and diversity may depend on the adjustment of the C/N ratio (Lucas et al., 2014). In general, low C/N ratio of amendments could have inhibitory effects on soil microbial activity, including decreasing microbial biomass and metabolites (Treseder, 2008). However, the effects of combined applications of different levels of nitrogen fertilizer and biochar (adjusting C/N ratio in soil) on soil microbial biomass and diversity are still unclear in soil reclamation of mining areas. Therefore, exploring most favorable ratio of combined nitrogen fertilizer and biochar for soil microbial biomass and diversity can provide a scientific basis for the sustainable restoration of soil in mining areas.

Qilian Mountains are important ecological security barrier in the western part of China (Du et al., 2015) and contain abundant hydropower and mineral resources (i.e., iron ore, copper ore, tungsten ore, coal mine). However, the local environment of the Qilian Mountains has been severely damaged due to illegal mining (i.e., unlicensed mining, mining of protected minerals) for economic benefits. The restoration and reconstruction of the damaged ecosystem in Qilian Mountains become an important task of environmental protection. However, there are still many difficulties in ecosystem restoration at field scale in this area due to high‐altitude, complex topography, large soil heterogeneity, cold, and changeable climate. Therefore, a short‐term soil incubation experiment (close to the local varying temperature) can overcome the above field difficulties and provide reference to land managers for application with damaged habitats in mining areas.

To relieve soil nutrient impoverishment and restore soil microbial diversity and mass caused by opencast mining in high‐altitude areas, soil reclamation was carried out with the addition of different amendments. On the basis of adding microbial agents to activate microbial activity, we aimed to determine the most favorable C/N ratio (adjusted by the level of nitrogen fertilizer and biochar) and select the most effective combination of amendments for promoting soil nutrients, microbial diversity, and mass in mining soil. We conducted a short‐term laboratory soil incubation experiment with a varying temperature for 12 weeks with 13 combined treatments by three amendments (biochar, nitrogen fertilizer, microbial agent). Our objectives were to (a) determine dynamics of soil physicochemical properties (pH, EC, soil organic carbon, total nitrogen, available nitrogen, available phosphorus, available potassium) over incubation time, (b) determine the effects of different amendments on microbial biomass (carbon and nitrogen), and composition and diversity of bacterial and fungal community; and (c) verify which C/N ratio adjusted by the combination of biochar and N level is more suitable for soil microbial growth.

## 2 MATERIALS AND METHODS

### 2.1 Soil sampling

On 16 August 2020, soil samples were collected from a tailing slope of an opencast coal mining area in Datan located in the Qilian Mountain (SE Gansu province) (36°50′54″N, 102°48′05″E, 2,650–2,660 m), China. The land use type around the sampling area is mainly natural grassland. The area has a typical semiarid and cold temperate climate, with mean temperature of about 16°C and mean precipitation of about 375 mm in the growing season (June to September). The soil type is Inceptisol under the USDA Soil Taxonomy system which is characterized by nutrient impoverishment, sandy to sandy loam texture, visible soil horizons, and heavy metal contents that do not exceed the standard. Five plots, 20 × 20 m^2^, were randomly located in a tailing slope of an opencast coal mining area, in which five soil samples (0–30 cm) were collected for each sample plot with a soil‐coring kit (20 cm in diameter), for a total of 25 soil samples with a total weight of 50 kg. All soil samples were thoroughly homogenized into one composite sample and sieved through a 4 mm sieve to discard coarse fragments prior to incubation experiments. Mine soil properties are shown in Table 1.

**TABLE 1:** Physicochemical properties in mine soil and biochar amendments (units: mg/kg)

Property	Mine soil	Biochar
pH	8.50	8.35
SOC	620	2.12 × 10^5^
TN	300	6,840
C/N	2.07	31.0
AN	18.23	62.71
AP	1.85	127.63
AK	51.87	2,970.0
Cu	9.92	–
Zn	66.7	–
Pb	23.5	–
Cd	0.21	–
Cr	44.9	–

Note

pH and C/N were dimensionless units.

Abbreviations: AK, available potassium; AN, available nitrogen; AP, available phosphorus; C/N, soil organic carbon/total nitrogen; SOC, soil organic carbon; TN, total nitrogen.

### 2.2 Amendments used and soil incubation

Three soil amendments were used for reclamation in mine soils, including biochar, nitrogen fertilizer, and microbial agent. (a) Biochar feedstock was crop residue (maize), which was air‐dried for 30 days and then was ground to pass a 2‐mm sieve. Then, the ground residues were pyrolyzed to form biochar in a muffle furnace with an increase at 5°C/min to 500°C for 2 hr. Biochar was ground to 250 µm for laboratory incubation. Details about biochar properties are shown in Table 1. (b) Nitrogen fertilizer was urea (46.67% N, CH_4_N_2_O). (c) Microbial agent (obtained from Beijing Danlu Biotechnology Co., Ltd) was mainly prepared with castor as a carrier. The carrier was placed in a polypropylene plastic bag and sterilized at 121°C for 1.5–2 hr, and then cooled. Subsequently, the carrier was inoculated with effective microorganism solution and put it in a 25°C incubator for 4–5 days after mixing. Since we aimed to activate the microbial activity in mine soil by the addition of microbial agent, thus, we selected the effective bacterial and fungal composition with a wide range of adaptability in extreme soil environments. The effective bacterial and fungal composition in the microbial agent (sequencing process according to Section 2.4) contained Ascomycota, *Basidiomycota,* *Chytridiomycota, Mortierellomycota* and *Proteobacteria, Actinobacteria*, *Bacteroidetes, Gemmatimonadetes* (the relative abundance >1%), and its effective amounts are up to >300 million g^−1^. The microbial agent contained the organic carbon content ≥50%, total N content is 1.11%, P_2_O_5_ is 1.04%, K_2_O is 0.31%, and the total contents of trace elements (i.e., B, Mn, and Zn) are 0.40%.

**FIGURE 1:**
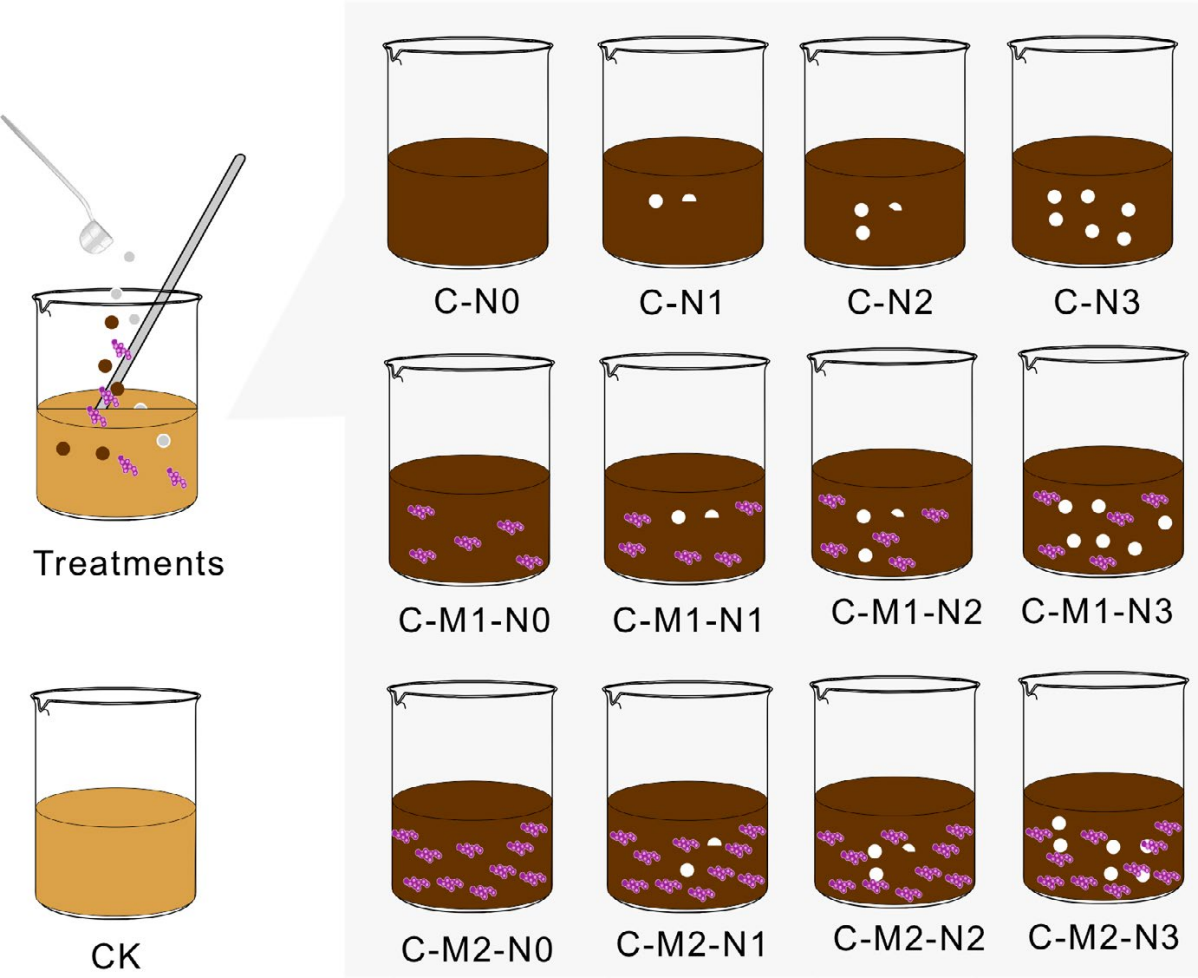
Soil reclamation treatments used for laboratory incubation. Red represents microbial agent levels. White represents nitrogen fertilizer levels. Light brown represents mine soil (reference). Brown represents mine soil with biochar

Biochar (C) was used for substrates in all treatments and was thoroughly mixed with mine soil at an application rate of 30 g carbon kg^−1^ soil, which was similar to the organic carbon content in the natural grassland soil in the sampling area. Based on the desired C/N ratios of 35:1, 25:1, and 12.5:1, nitrogen fertilizer (N) was added at three levels of 0.86 (N_1_), 1.2 (N_2_), and 2.4 (N_3_) g N/kg soil, respectively. Microbial agents (M) and 20 g of distilled water were thoroughly mixed and sprayed into the mine soil at a dose of 0.4 (M_1_) and 0.8 (M_2_) g/kg soil. Thirteen different treatments with three replicates per treatment were applied to the soil samples: C‐N_0_, C‐N_1_, C‐N_2_, C‐N_3_; C‐M_1_‐N_0_, C‐M_1_‐N_1_, C‐M_1_‐N_2_, C‐M_1_‐N_3_; C‐M_2_‐N_0_, C‐M_2_‐N_1_, C‐M_2_‐N_2_, C‐M_2_‐N_3_, and unamended mine soil was used as control (CK) (Figure 1).

Laboratory incubation was carried out with 1,000 g of mine soil in a 2‐L beaker under aerobic and dark conditions for 90 days in a varying temperature incubator (JYL‐253, Jiayu, Shanghai, China), at a constant soil moisture of 50% of water holding capacity and a varying temperature which gradually increased from 5 to 22°C for the first 12 hr, and then decreased from 22 to 5°C for the last 12 hr (close to the local summer temperature condition). Soils were sampled at 15°C incubation temperature(close to the local annual average temperature condition) to monitor pH, soil organic carbon (SOC), total nitrogen (TN), available nitrogen (AN), available phosphorus (AP), available potassium (AK), microbial biomass carbon (MBC), and microbial biomass nitrogen (MBN) at 0, 5, 12, 45, and 90 days of experiment. The first sampling point (day 0) was collected just after soil sampling. To observe the effect of different treatment and amendments on the structure and diversity of microbial communities, bacterial and fungal communities were assayed at the end of the incubation experiment.

### 2.3 Soil physicochemical and microbial biomass analyses

Amended soil samples were divided into two parts: One part was air‐dried in preparation for soil elemental analyses, and the other was stored at 4°C in a refrigerator for microbial measurements. Air‐dried soil sample was shaken on a 2‐mm sieve for the measurements of pH, SOC, TN, AN, AP, and AK. Detailed soil pH, SOC, TN, and AN measurements were described in Chen, He et al. (2020). AP and AK measurements were described in Bray and Kurtz (1966) and Jackson (1973).

Soil samples stored at 4°C were measured for MBC and MBN using chloroform fumigation and extraction method (Vance et al., 1987). Briefly, 10 g of oven‐dry soil was fumigated with chloroform in the dark for 48 hr after which C and N of fumigated and nonfumigated (control) samples was extracted with 0.5 ml K_2_SO_4_, and then total dissolved organic C was determined on an organic carbon analyzer (Shimadzu Model TOC), while total extractable N was quantified with a flow‐injection instrument. After values in nonfumigated were subtracted from those of fumigated samples, a Kec/Ken factor of 0.45 and 0.54 was applied for MBC both MBN.

### 2.4 Microbial abundance and community structure

#### 2.4.1 DNA extraction, PCR amplification, and sequencing

Soil biological samples representing different treatments were frozen at −80°C for further DNA analysis. DNA was directly extracted using Power Soil kit 152 (MoBio Laboratories, Carlsbad, CA, USA) following the manufacturer's instructions for specific amplification and high‐throughput sequencing (Cota‐Sánchez et al., 2006).

The V3‐V4 region of bacterial 16S rRNA gene was amplified by a polymerase chain reaction (PCR) using the primer 341F (5′‐CCTAYGGGRBGCASCAG‐3′) and 806R (5′‐GGACTACNNGGGTATCTAAT‐3′). PCRs were carried out in a 25 µl mixture with three replicates per DNA sample, containing 5 μl of Q5 reaction buffer (5×), 5 μl of Q5 High‐Fidelity GC buffer (5×), 0.25 μl of Q5 High‐Fidelity DNA Polymerase (5 U/μl), 2 μl (2.5 mM) of dNTPs, 1 μl (10 µM) of each Forward and Reverse primers, 2 μl of DNA Template, and 8.75 μl of dd H_2_O (Zhang et al., 2020). The fungal ITS rRNA genes were amplified with ITS1F(5′‐CTTGGTCATTTAGAGGAAGTAA‐3′)/ITS2R(5′‐GCTGCGTTCTTCATCGATGC‐3′) primers. PCRs were carried out in a 25 µl mixture with three replicates per DNA sample, containing 2 μl of 10× Buffer, 2 μl of 2.5 mM dNTPs, 0.8 μl of each Primer (5 μM), 0.2 μl of rTaq Polymerase, and 10 ng Template DNA. PCR amplification was performed under the following cycling conditions: initial denaturation at 98°C for 2 min, followed by 25 cycles consisting of denaturation at 98°C for 15 s, annealing at 55°C for 30 s, and extension at 72°C for 30 s, with a final extension of 5 min. All PCR amplifications were performed in triplicate and then combined. PCR amplicons were then pooled in equimolar concentrations on a 1% agarose gel, and purified PCR products were recovered using a Gel Extraction Kit (Omega Bio‐Tek, Norcross, GA, USA). High‐throughput sequencing of the PCR products was performed on an Illumina Miseq platform (Miseq PE250) (McGuire et al., 2013).

#### 2.4.2 Sequencing data processing

Raw sequence data were quality‐filtered, and chimera was checked using the QIIME software (version 1.8.0) to remove reads containing more than 10% unknown nucleotides and reads were fewer than 50% of all bases had quality values (*Q*‐values) > 20 (Caporaso et al., 2010). Operational taxonomic units (OTUs) were clustered with a sequence threshold of 97% similarity by UPARSE39, and representative sequences of OTUs were picked up simultaneously. The tag sequence with the highest abundance within each cluster was selected as the representative sequence. The taxonomic assignment of 16S rRNA sequences was determined using the bacterial SSUrRNA reference database with the Mothur and SILVA (http://www.arb‐silva.de/) classifier and the taxonomic assignment of ITS sequences was determined using the Unite reference database (http://unite.ut.ee/index.php) with the Ribosomal Database Project (RDP) classifier at a 97% level (Edgar et al., 2011).

### 2.5 Statistical analyses

Significant differences of treatments on soil physicochemical properties, microbial biomass, and alpha diversity indices for microbial communities were detected by one‐way analysis of variance (ANOVA) followed by a least significant difference (LSD) multiple comparison using SPSS version 17.0 (SPSS Inc., Chicago, IL, United States). Meanwhile, in order to visually display the effects of only adding one and the combination of two or three amendments on soil physicochemical properties and microbial biomass, we classified thirteen treatments into five treatments for further one‐way analysis: CK, C (C‐N0), C‐N (C‐N1, C‐N2, C‐N3), C‐M (C‐M1‐N0, C‐M2‐N0), and C‐M‐N (C‐M1‐N1, C‐M1‐N2, C‐M1‐N3; C‐M2‐N1, C‐M2‐N2, C‐M2‐N3). General linear model (GLM) analysis with repeated measures was used to analyze the integrative effects of incubation time and treatments on soil physicochemical properties, microbial biomass. Heatmaps were generated using Omicsmart, a dynamic real‐time interactive online platform for data analysis (http://www.omicsmart.com). Alpha diversity indices (including Chao1, Shannon, and Simpson) were calculated using QIIME software. According to a Bray–Curtis similarity matrix, principal coordinates analysis (PCoA) was conducted to analyze the overall differences in bacterial and fungal community structures among different treatments. In addition, redundancy analysis (RDA) was aimed to assess the effects of soil physicochemical properties and microbial biomass on bacterial and fungal community composition at phylum level and to extract key soil properties driving the variability in bacterial and fungal community composition after the addition of amendments.

## 3 RESULTS

### 3.1 Effect of amendments on soil physicochemical properties

Amended treatments and incubation time had significant effects on physicochemical properties, and with significant interactions between the two factors (*p* < .001). During the entire incubation period, soil pH was significantly lower after addition of N fertilizer; a significant increase in SOC, TN, AN, AP, and AK contents in all amended treatments than CK treatment (Table 2, Table S1).

**TABLE 2:** Soil physicochemical properties, microbial mass, and its ratio in different treatments

Soil properties	CK	C	C‐N	C‐M	C‐M‐N	Treatment		Time		Interaction	
						*F*	*p*	*F*	*p*	*F*	*p*
pH	8.64 ± 0.01a	8.71 ± 0.02a	8.47 ± 0.04b	8.72 ± 0.02a	8.44 ± 0.02b	37.03	***	23.73	***	7.39	***
SOC	0.71 ± 0.01c	11.92 ± 0.17b	11.56 ± 0.09b	12.57 ± 0.14a	12.40 ± 0.08a	4,277.37	***	68.43	***	4.16	***
TN	0.37 ± 0.01c	1.15 ± 0.12b	1.45 ± 0.76a	1.44 ± 2.31a	1.63 ± 2.71a	59.79	***	149.85	***	29.35	***
AN	17.38 ± 0.30d	29.86 ± 2.16c	33.18 ± 0.93c	43.28 ± 3.63b	55.16 ± 1.88a	129.34	***	38.61	***	10.01	***
AP	1.90 ± 0.01c	13.79 ± 0.83b	17.37 ± 0.61a	14.81 ± 0.53b	17.75 ± 0.61a	55.09	***	6.77	***	1.44	***
AK	51.65 ± 0.61c	166.48 ± 12.36b	148.63 ± 5.03b	210.09 ± 10.21a	212.23 ± 7.63a	70.66	***	22.96	***	3.08	***
MBC	49.2 ± 0.77d	192.64 ± 8.88c	204.54 ± 6.52c	231.73 ± 7.66b	254.34 ± 6.87a	88.57	***	14.88	***	1.04	–
MBN	5.15 ± 0.12d	21.25 ± 1.00c	22.86 ± 0.45c	26.10 ± 0.76b	29.93 ± 0.58a	162.93	***	10.84	***	1.10	–
MBC/MBN	9.60 ± 0.16a	9.10 ± 0.19a	8.89 ± 0.17a	8.89 ± 0.18a	8.50 ± 0.16b	3.67	**	3.83	**	0.8	–

Note

Values are means ± standard error. Different letters indicate significant differences: *p < .05, **p < .01, ***p < .001. “–” indicate no significant differences. CK, C, C‐N, C‐M, and C‐M‐N represent unamended mine soil, biochar, biochar + N fertilizer, biochar + microbial agent, and biochar + N fertilizer + microbial agent.

**FIGURE 2:**
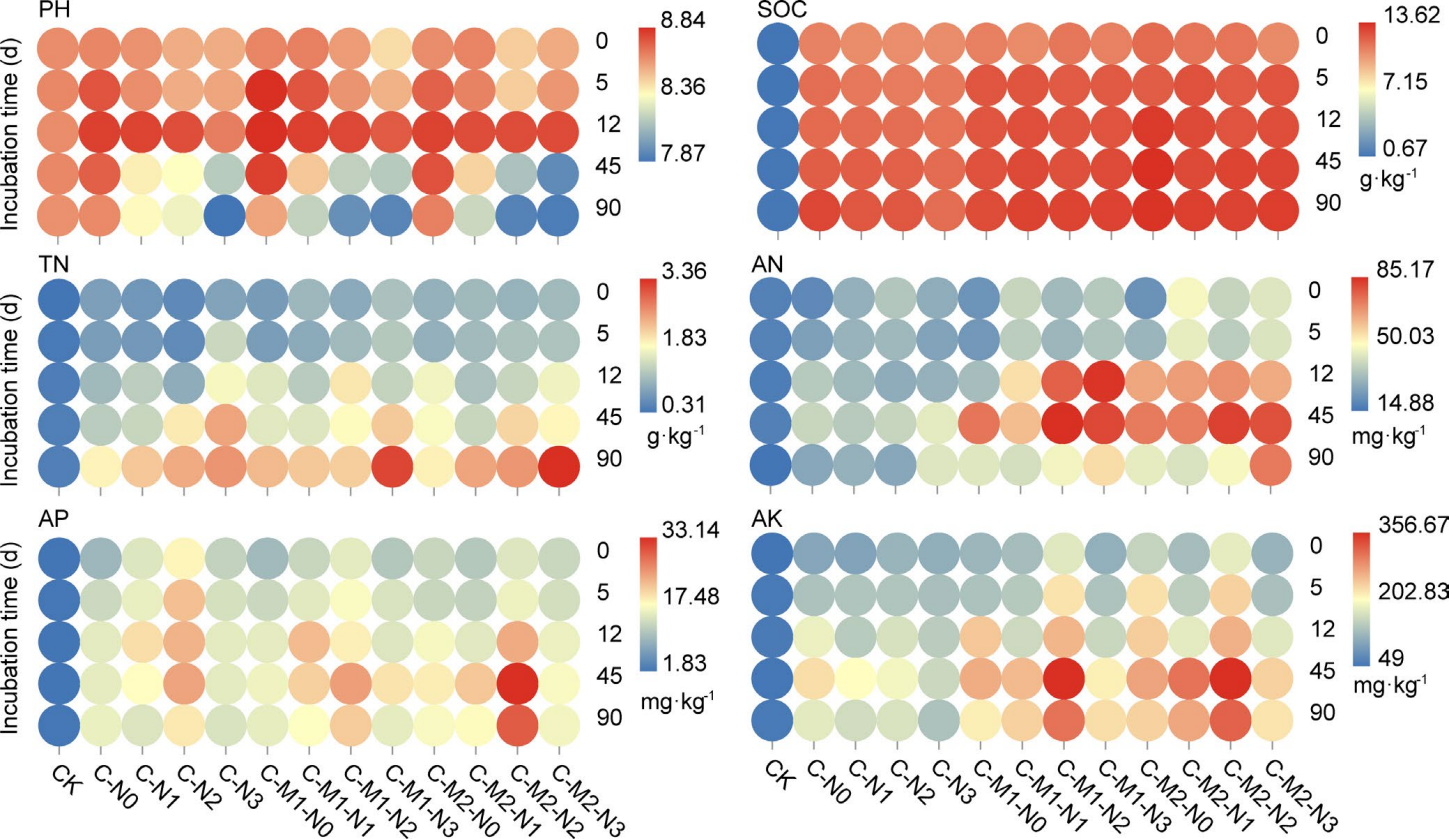
Heatmap analysis of the relationships between soil physicochemical properties and incubation time in different amended treatments. The point denotes the average value of each property at each incubation time (*n* = 3). AK, available potassium (mg/kg); AN, available nitrogen (mg/kg); AP, available phosphorus (mg/kg); SOC, soil organic carbon (g/kg); TN, total nitrogen (g/kg)

Specifically, pH decreased more in N3 level, ordered by C‐M2‐N3, C‐M1‐N3, and C‐N3. SOC had higher contents in M2 level, ordered by C‐M2‐N0, C‐M2‐N2, C‐M2‐N1, and C‐M2‐N3. TN and AN contents had higher contents in N3 level, both ordered by C‐M2‐N3, C‐M1‐N3, and C‐N3. N2 level had higher AP and AK contents, in which C‐M2‐N2 and C‐M1‐N2 treatments supported the higher value (Table S1, Figure 2). With the increasing incubation time (Figure 2), pH increased up to day 12 and then decreased during the remaining incubation time (day 12 to 90) in all amended treatments. AN, AP, and AK increased significantly in all amended treatments up to day 45 and then decreased during the remaining incubation time (day 45 to 90). In addition, there was an increase in SOC and TN contents with the increasing incubation time in all amended treatments.

### 3.2 Effect of amendments on soil MBC, MBN, and MBC/MBN

Amended treatments and incubation time had significant effects on MBC, MBN (*p* < .001), and MBC/MBN (*p* < .01), but no significant interactions between the two factors. During the entire incubation period, MBC and MBN in all amended treatments were significantly higher than those in CK treatment, while MBC/MBN only in C + M + N treatments were significantly lower than that of CK treatment (Table 2, Table S1).

**FIGURE 3:**
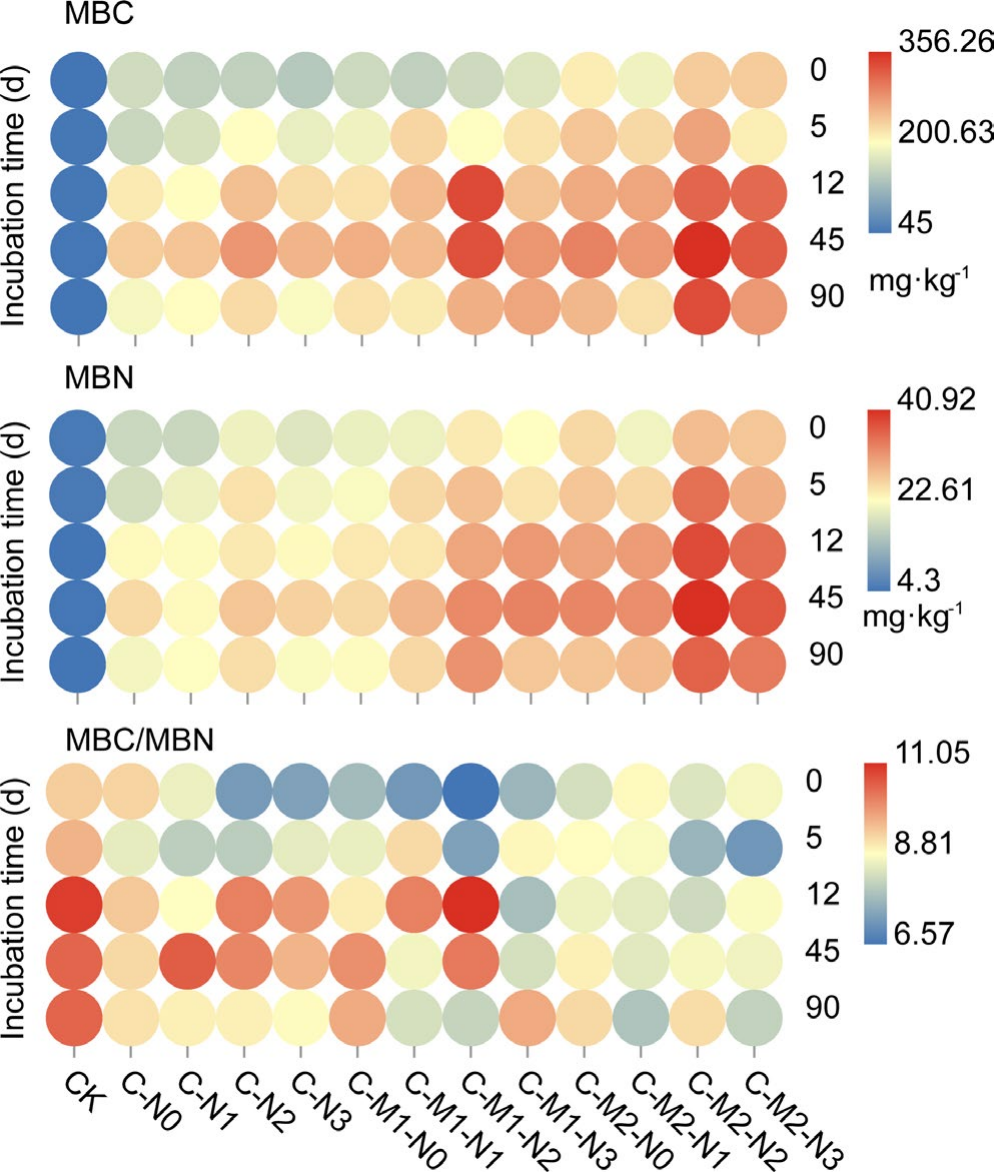
Heatmap analysis of the relationships between soil microbial biomass and incubation time in different amended treatments. The point denotes the average value of each property at each incubation time (*n* = 3). MBC, microbial biomass carbon (mg/kg); MBC/MBN, microbial biomass carbon/microbial biomass nitrogen; MBN, microbial biomass nitrogen (mg/kg)

Specifically, MBC and MBN contents both increased more after M addition (in C‐M‐N and C‐M treatments), ordered by C‐M2‐N2, C‐M2‐N3, and C‐M1‐N2. MBC/MBN decreased more in N2 levels, in which C+M2+N2 supported the lowest value (Table S1, Figure 3). With the increasing incubation time (Figure 3), MBC, MBN, and MBC/MBN in CK treatment remained almost unaltered with the average value of 42 mg/kg, 5 mg/kg, and 9.52, respectively. MBC and MBN (*p* < .01) increased significantly up to day 45 and then decreased during the remaining incubation time (day 45 to 90). In all amended treatments, MBC/MBN exhibited significant changes with incubation time but no obvious regularity.

### 3.3 Diversity and composition of soil bacterial community

A total of 1,532,205 bacterial sequences were obtained from the complete dataset, of which 13,769 bacterial OTUs belonged to 33 phyla, 257 classes, 257 orders, 475 families, and 1,000 genera. The rarefaction curves of bacteria showed clear asymptotes, which indicated a near‐complete and true sampling of the community. The dominant phyla (relative abundance >1%) were Proteobacteria (48.26%–82.32%), Actinobacteria (16.59%–4.09%), Bacteroidetes (4.49%–15.47%), Firmicutes (0.55%–9.20%), Gemmatimonadetes (1.76%–8.18%), Chloroflexi (0.89%–5.21%), Patescibacteria (0.87%–5.17%), and Acidobacteria (0.09%–2.72%), together accounting for >98% of bacterial sequences across all samples (Table 3, Table S2). Notably, the relative abundance of Bacteroidetes increased significantly in C‐M‐N treatments (*p* < .001) (Table S2), in which C‐M2‐N2 level increased the highest value (Table 3). Firmicutes also increased significantly in C‐M‐N treatments (*p* < .01), in which N1 level increased the higher value (ordered by C‐M2‐N1, C‐M1‐N1). However, the relative abundance of Acidobacteria decreased significantly (*p* < .001) with addition of N fertilizer, in which N1 level (ordered by C‐M2‐N1, C‐N1, C‐M1‐N1) decreased obviously (Table 3).

**TABLE 3:** Relative abundance of the dominant bacterial and fungal phyla (relative abundance >1%) in different treatments

	Taxon	CK	C‐N0	C‐N1	C‐N2	C‐N3	C‐M1‐N0	C‐M1‐N1	C‐M1‐N2	C‐M1‐N3	C‐M2‐N0	C‐M2‐N1	C‐M2‐N2	C‐M2‐N3
Bacteria	Proteobacteria	73.98 ± 14.44ab	62.69 ± 4.72bcd	61.00 ± 5.22bcd	71.20 ± 1.83abc	69.21 ± 2.18bcd	83.58 ± 2.96a	55.65 ± 3.52d	63.77 ± 0.62bc	66.80 ± 1.86bcd	58.05 ± 2.78cd	49.54 ± 2.50e	56.09 ± 2.27d	67.13 ± 2.30bcd
	Actinobacteria	11.19 ± 8.27a	14.78 ± 2.61a	16.31 ± 4.54a	11.08 ± 0.43a	13.47 ± 0.89a	8.89 ± 0.74a	11.41 ± 1.45a	11.16 ± 0.88a	9.1 ± 1.37a	7.78 ± 3.05a	14.69 ± 1.93a	9.83 ± 1.20a	8.83 ± 0.88a
	Bacteroidetes	4.98 ± 1.23f	6.03 ± 0.67ef	9.29 ± 0.92cde	10.06 ± 1.72cd	7.31 ± 1.58def	4.59 ± 0.88f	12.15 ± 1.95bc	11.97 ± 0.99bc	9.09 ± 1.00cde	8 ± 0.53def	15.1 ± 2.26b	19.34 ± 1.11a	9.8 ± 1.01cde
	Gemmatimonadetes	2.31 ± 1.39efg	4.21 ± 0.94cdef	4.5 ± 0.51cde	3.97 ± 0.42cdefg	2.69 ± 0.36defg	1.76 ± 0.64g	6.87 ± 1.47a	5.05 ± 0.24bc	4.5 ± 0.16cde	4.75 ± 0.76bcd	1.96 ± 0.36g	8.18 ± 0.87ab	4.35 ± 0.16cde
	Acidobacteria	1.37 ± 0.88bcd	2.73 ± 0.81a	0.10 ± 0.02e	0.34 ± 0.16de	0.69 ± 0.08cde	1.94 ± 0.51ab	0.09 ± 0.02e	0.4 ± 0.05de	0.49 ± 0.07de	1.76 ± 0.25abc	0.06 ± 0.04e	0.09 ± 0.05e	0.38 ± 0.10de
	Chloroflexi	1.89 ± 1.23ab	2.32 ± 0.49ab	1.06 ± 0.21b	0.91 ± 0.11b	1.12 ± 0.20b	2.89 ± 0.25a	2.12 ± 0.20ab	1.99 ± 0.10ab	0.87 ± 0.19b	3.21 ± 1.36a	2.53 ± 0.54ab	2.33 ± 0.40ab	0.94 ± 0.28b
	Patescibacteria	1.23 ± 0.59c	5.17 ± 2.75a	2.02 ± 0.56bc	0.87 ± 0.24c	3.77 ± 0.76abc	1.5 ± 0.66c	1.68 ± 0.09bc	0.96 ± 0.23c	4.52 ± 1.81ab	1.91 ± 0.28bc	1.79 ± 0.35bc	1.01 ± 0.36c	1.6 ± 0.23c
	Firmicutes	0.76 ± 0.16de	0.85 ± 0.31de	4.3 ± 0.98b	0.71 ± 0.27de	0.55 ± 0.05e	1.52 ± 0.60cde	4.29 ± 1.03b	3.46 ± 0.78bc	3.11 ± 0.39bcd	4.29 ± 0.34b	9.2 ± 1.85a	3.47 ± 1.12bc	5.26 ± 0.95b
Fungal	Ascomycota	79.06 ± 01.78a	85.66 ± 4.46a	79.78 ± 12.04a	84.87 ± 4.86a	79.93 ± 03.46a	70.23 ± 11.6a	73.33 ± 3.62a	76.05 ± 4.17a	76.54 ± 21.40a	72.54±8a	75.55 ± 7.73a	69.63 ± 4.22a	68.93 ± 9.77a
	unclassified_Fungi	6.91 ± 1.82a	3.61 ± 1.03a	17.99 ± 1.94a	7.95±0.27a	5.32 ± 1.40a	3.45 ± 2.53a	11.66 ± 2.68a	8.54 ± 3.28a	9.52 ± 2.74a	18.34 ± 7.33a	8.45 ± 0.74a	7.36 ± 0.69a	15.77 ± 0.72a
	Basidiomycota	6.55 ± 1.04bcd	4.07 ± 0.34a	1.64 ± 0.53d	3.47 ± 0.70cd	9.21 ± 2.61ab	13.47 ± 0.92a	4.74 ± 2.52bcd	4.96 ± 1.44bcd	7.21 ± 2.42bc	13.7 ± 1.57a	6.18 ± 3.83bcd	3.95 ± 1.19cd	5.12 ± 0.26bcd
	unidentified	3.15 ± 0.80bc	6.04 ± 2.93bc	0.45 ± 0.14c	3.38 ± 2.45bc	3.6 ± 1.17bc	2.53 ± 0.77bc	9.97 ± 1.86abc	9.35 ± 0.93abc	6.57 ± 3.30bc	3.51 ± 0.87bc	9.32 ± 1.10abc	18.08 ± 2.23a	9.91 ± 3.08abc
	Chytridiomycota	2.93 ± 0.93a	0.2 ± 0.19b	0	0	0.26 ± 0.01b	0	0.03 ± 0.02b	0.35 ± 0.20b	0	0.3 ± 0.20b	0.03 ± 0.02b	0	0

Note

Values are means ± standard error. Different letters indicate significant differences in different treatments (*p* < .05).

* *p* < .05

** *p* < .01

*** *p* < .001.

Alpha diversity estimated by Chao1 estimator, Shannon, and Simpson indices showed significant differences in species richness and diversity of soil bacterial community between different treatments (*p* < .05). Chao1 estimator was significantly higher in C (C‐N0), C + M (C‐M1‐N0, C‐M2‐N0), and C + M + N2 (C‐N2, C‐M1‐N2, C‐M2‐N2) treatments than in CK treatment (*p* < .05), in which N2 level treatment supported higher value (highest in C‐M2‐N2 treatment). Simpson and Shannon indices were significantly higher in all amended treatments especially at the N2 level (*p* < .05), but no significant difference was observed between amended treatments (Table 4).

**TABLE 4:** Richness and diversity indices of bacterial (a) and fungal (b) communities in different treatments

Treatments	Bacteria			Fungi		
	Chao1	Simpson	Shannon	Chao1	Simpson	Shannon
CK	624.22 ± 254.92e	0.81 ± 0.1b	5.25 ± 1.52b	50.03 ± 7.48e	0.85 ± 0.02a	3.76 ± 0.15b
C‐N0	**877.43 ± 11.76bcd**	0.97 ± 0.02a	7.91 ± 0.52a	**139.13 ± 28.19cd**	0.92 ± 0.004a	4.73 ± 0.17ab
C‐N1	639.1 ± 56.42e	0.98 ± 0.003a	7.49 ± 0.10a	91.30 ± 3.31de	0.9 ± 0.006a	3.99 ± 0.06ab
C‐N2	**1,045.32 ± 38.75ab**	0.98 ± 0.003a	7.51 ± 0.16a	73.95 ± 16.67e	0.93 ± 0.03a	4.06 ± 0.29ab
C‐N3	740.89 ± 111.03de	0.98 ± 0.004a	7.33 ± 0.27a	54.65 ± 5.42e	0.94 ± 0.004a	4.50 ± 0.07ab
C‐M1‐N0	**873.89 ± 30.20cd**	0.94 ± 0.05a	7.23 ± 0.83a	58.84 ± 10.82e	0.86 ± 0.02a	3.79 ± 0.06b
C‐M1‐N1	804.79 ± 14.26cd	0.99 ± 0.0008a	7.88 ± 0.05a	107.45 ± 27.89de	0.88 ± 0.03a	4.14 ± 0.40ab
C‐M1‐N2	**1,080.22 ± 126.82ab**	0.99 ± 0.001a	7.96 ± 0.05a	102.69 ± 7.93de	0.94 ± 0.008a	**5.25 ± 0.26a**
C‐M1‐N3	720.54 ± 150.13e	0.99 ± 0.001a	7.58 ± 0.06a	105.47 ± 1.97de	0.96 ± 0.019a	4.89 ± 0.16ab
C‐M2‐N0	**903.53 ± 49.69abc**	0.99 ± 0.002a	7.57 ± 0.07a	**308.23 ± 26.87a**	0.88 ± 0.04a	4.56 ± 0.24ab
C‐M2‐N1	874.41 ± 16.28bcd	0.99 ± 0.003a	7.54 ± 0.21a	**170.49 ± 20.52c**	0.91 ± 0.03a	4.77 ± 0.62ab
C‐M2‐N2	**1,233.68 ± 46.97a**	0.99 ± 0.004a	7.59 ± 0.06a	**233.08 ± 39.81b**	0.95 ± 0.02a	**5.58 ± 0.32a**
C‐M2‐N3	854.47 ± 6.21cd	0.98 ± 0.003a	7.5 ± 0.18a	**106.32 ± 30.47de**	0.94 ± 0.008a	4.89 ± 0.08ab

Note

Values are means ± standard error. Different letters indicate significant differences. Bold values represent significantly higher treatments than CK.

* *p* < .05

** *p* < .01

*** *p* < .001.

**FIGURE 4:**
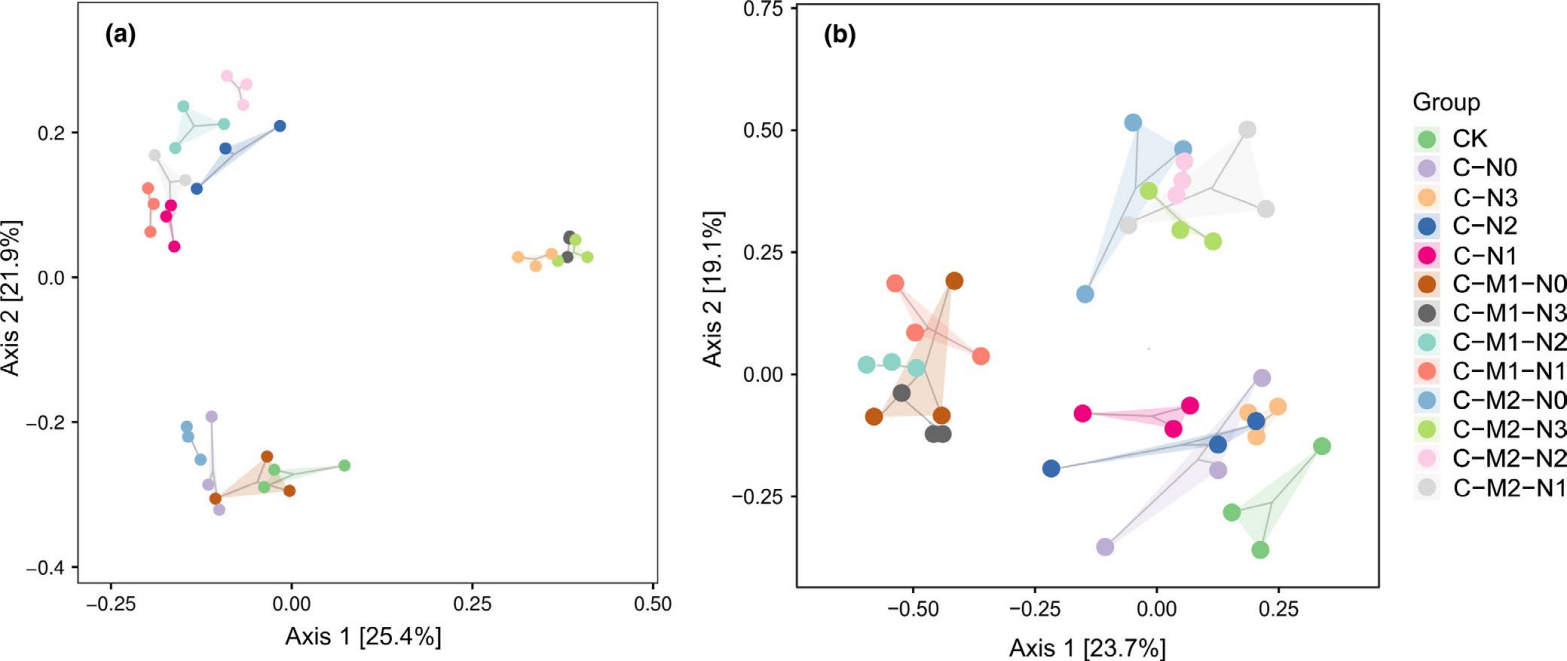
Principal coordinate analysis (PCoA) of bacterial (a) and fungal (b) community composition based on Bray–Curtis distances. Values at axes 1 and 2 are the percentages that can be explained by the corresponding axis

PCoA analysis based on Bray–Curtis distances accounted for 47.3% of total variance among bacterial communities, with axes 1 and 2 explaining 25.4 and 21.9% of the variance, respectively (Figure 4a). PCoA analysis showed that bacterial communities were divided into three major groups. Treatments with N1 and N2 level (C‐N1, C‐N2, C‐M1‐N1, C‐M1‐N2, C‐M2‐N1, and C‐M2‐N2) tended to group together, N3 level (C‐N3, C‐M1‐N3, and C‐M2‐N3) clustered into another group, and treatments without N addition (C‐N0, C‐M1‐N0, and C‐M2‐N0) grouped together with CK (*R* ^2^ = .555 > .5, *p* < .001; PERMANOVA, Test statistic = 5.0189, *p* = .001). Overall, three groups exhibited significant differences in bacterial community composition and were separated mainly by N level.

### 3.4 Diversity and composition of soil fungal community

A total of 2,625,602 fungal sequences were obtained from the complete dataset, of which 2,446 fungal OTUs belonged to 12 phyla, 36 classes, 89 orders, 226 families, and 417 genera. The rarefaction curves of fungal showed clear asymptotes, which indicated a near‐complete and true sampling of the community. The dominant phyla were in the ranking order: Ascomycota (85.66%–51.53%), Basidiomycota (31.75%–1.64%), unclassified Fungi (18.34%–3.45%), unidentified (18.07%–2.53%), and Chytridiomycota (2.93%–0.01%), together accounting for >98% of fungal sequences across all samples (Table 3, Table S2). Notably, the relative abundance of Chytridiomycota (*p* < .05) decreased significantly with addition of amendments, in which C + M + N treatments decreased the most. No significant differences were observed in Ascomycota (except for unclassified Fungi and unidentified) at all amended treatments, and Ascomycota decreased in C + M and C + M + N addition (especially in C‐M2‐N3). Basidiomycota increased significantly in C + M treatments (C‐M2‐N0, C‐M1‐N0) (Table 3, Table S2).

Alpha diversity estimated by Chao1 estimator and Shannon indices showed significant differences in species richness and diversity of soil fungal community between different treatments (*p* < .05). Chao1 estimator was significantly higher in C (C‐N0), C+M2+N (C‐M2‐N0, C‐M2‐N1, C‐M2‐N2, C‐M2‐N3) treatments than in CK treatment (*p* < .05), in which M2 level supported higher value. Simpson indices had no significant difference between amended and CK treatments. Shannon indices were significantly higher in C+M1+N2 and C‐M2‐N2 treatments (Table 4).

PCoA analysis based on Bray–Curtis distances accounted for 42.8% of total variance among the fungal communities, with axes 1 and 2 explaining 23.7 and 19.1% of the variance, respectively (Figure 4b). PCoA analysis showed that fungal communities also were divided into three major groups. All M2‐level treatments (C‐M2‐N0, C‐M2‐N1, C‐M2‐N2, C‐M2‐N3) tended to group together, M1 level (C‐M1‐N0, C‐M1‐N1, C‐M1‐N2, C‐M1‐N3) clustered into another group, and treatments without M addition (C‐N0, C‐N1, C‐N2, C‐N3) were grouped together with CK (*R* ^2^ = .504 > .5, *p* < .001; PERMANOVA, Test statistic = 2.203, *p* = .001). Overall, three groups exhibited significant differences in fungal community composition and were separated mainly by M level.

### 3.5 Relationships between soil bacterial, fungal community, and soil properties

**FIGURE 5:**
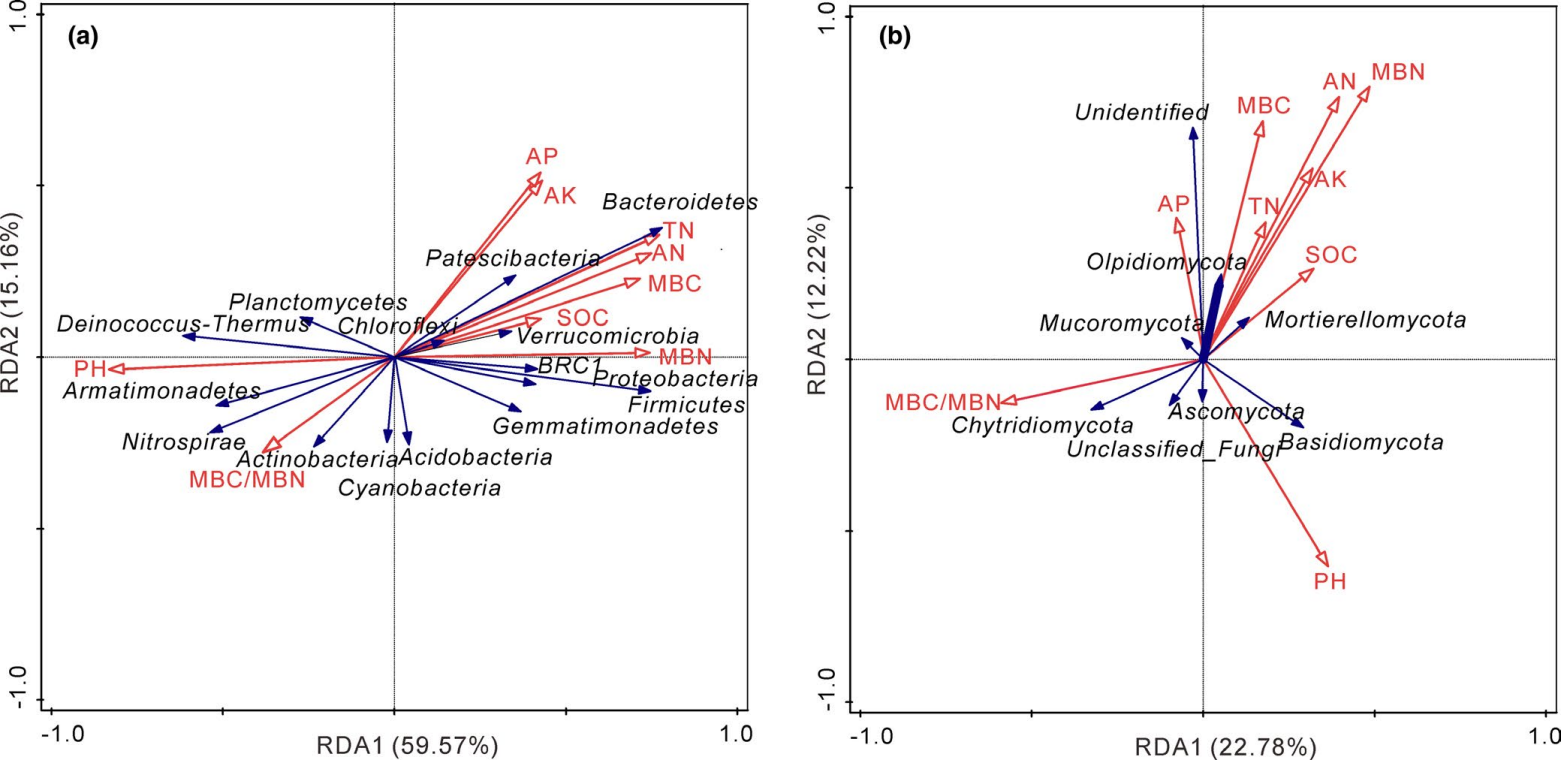
Redundancy analysis (RDA) identifying the relationships between bacterial (a) and fungal (b) phyla and soil properties in different treatments. Values at axes 1 and 2 are the percentages explained by the corresponding axis

Redundancy analysis (RDA) depicts the relationships between dominant phyla of soil bacterial and fungal communities (relative abundance >1%) and nine selected soil physicochemical properties and microbial biomass (Figure 5). RDA showed that pH (*r* ^2 ^= .91, *p* = .001), TN (*r* ^2 ^= .76, *p* = .035), and MBN (*r* ^2 ^= .73, *p* = .005) were the most significant environmental factors explaining variability in bacterial community composition, with the first two axes accounting for 59.57 and 15.16% of the total variation (74.73%), respectively (Figure 5a). Proteobacteria, Firmicutes, and Bacteroidetes were negatively correlated with pH and MBC/MBN, while significantly positively correlated with other properties; Acidobacteria were negatively correlated with pH, TN, and AN. RDA showed that MBN (*r* ^2 ^= .84, *p* = .001), pH (*r* ^2 ^= .76, *p* = .001), AN (*r* ^2 ^= .78, *p* = .001), and MBC (*r* ^2 ^= .79, *p* = .001) were the most significant environmental factors explaining variability in fungal community composition, with the first two axes accounting for 22.78% and 12.22% of the total variation (35.0%), respectively (Figure 5b). Ascomycota and Chytridiomycota showed a positive correlation with pH and MBC/MBN, and a negative correlation with other soil properties; Basidiomycota showed a positive correlation with pH, SOC, MBN, and AK.

## 4 DISCUSSION

### 4.1 Changes in soil physicochemical properties

Our results demonstrated that the amended treatments greatly affected soil physicochemical properties. We believed that the change of each soil property was likely to be affected by a single amendment or a combination of amendments that were added in our experiment. In general, the effectiveness of biochar in improving soil pH depends not only on the alkalinity of biochar itself but also on the carbonates (MgCO_3_, CaCO_3_) and organic acid radicals (–COO–) formed during the formation of biochar (Clough et al., 2013). In our study, the application of biochar alone did contribute to an increase in soil pH, but no significant increase has been observed, which was likely due to the fact that biochar can regulate the pH of acid soil better than that of alkaline soil (Ok et al., 2015). However, N fertilizer addition significantly (*p* < .001) decreased soil pH by the end of incubation period. However, we found that soil pH was significantly lower after N addition (*p* < .001) (especially in N3 level) than in CK treatment over the incubation period. This confirmed that the higher content of N addition may be an effective method for pH neutralization in alkaline soils in a certain range, as also observed in other recent studies (Pan et al., 2020; Wang et al., 2020). The addition of N fertilizers may lead to decreases in soil pH due to the oxidation and nitrification of ammonia (Geisseler & Scow, 2014).

We observed a significant increase in SOC contents in all amended treatments than CK treatment over the incubation time. The increased SOC was likely due to the release of high OC contents from biochar into the soil. That biochar increases SOC contents has already been demonstrated in many studies (Agegnehu et al., 2016; Forján et al., 2017). In addition, we found that the addition of microbial agents, especially at high amounts (M2 level), also lead to a higher increase in SOC contents, indicating that the addition of microbial agents to the mine soil in this study improved SOC contents; this may be attributed to two reasons: First, the microbial agent itself contains more SOC contents, and second, the addition of microbial agents can produce a variety of enzymes (i.e., catalase, peroxidase, urease) by their life activities to promote the synthesis of soil organic matter (Song et al., 2007). TN also had significant increases in amended treatments, especially in N3 level (highest in C‐N3). This result indicated that the increase in TN may originate from high contents of N addition to mine soil. Subsequently, we subtracted TN contents introduced by N fertilizer and biochar, and TN contents were still higher in amended treatments; we attributed this to nitrogen retention in biochar. Certainly, several mechanisms have explained the apparent retention of N in biochar‐amended soils and the reduction in N leaching (i.e., adsorption of NH_3_ or organic‐N onto biochar) (Clough et al., 2013). In our study, a significant increase in AN contents in all amended treatments than CK treatment similar to TN, and the increase was most obvious at the N3 level (especially in C‐M2‐N3 treatment). The first reason may be the higher contents of N fertilizer addition and N contents contained in the biochar itself. Another possible reason could be that higher microbial activity due to the addition of M contributed to N mineralization and increased AN content (Ahirwal & Maiti, 2018).

AP and AK also showed a significant increase in all amended treatments and also exhibited higher contents both at the N2 level, which C‐M2‐N2 and C‐M1‐N2 treatments supported the higher value. Firstly, studies have shown that the addition of biochar can increase AP contents in soils because biochar itself contains large amounts of P with higher effectiveness (Agegnehu et al., 2016; Rafael et al., 2020). Secondly, AP and AK exhibited higher contents in N2 levels, indicating that the appropriate N sources for microorganisms can activate the activities of microorganisms, which in turn promotes the activation and decomposition of insoluble substances in the soil. More importantly, we found AP and AK contents increased with increasing M contents in N2 level. A possible explanation for this is that the higher contents of M added into the soil can reduce the fixation of P and K and improve the availability of soil P and K; also, the number of soil microorganisms after the higher contents of M addition increased significantly, which promoted the mineralization of P and the conversion of organophosphorus to AP due to the release of P affected by bacteria (i.e., Bacillus subtilis) (Hu et al., 2009). Above all, relatively higher contents of N and M addition based on biochar in the short‐term can improve soil quality by neutralizing soil pH and increasing soil nutrient contents.

### 4.2 Changes in soil MBC, MBN, and MBC/MBN

Our results demonstrated that MBC and MBN had a significant increase in amended treatments over the incubation period. In general, the variability in MBC and MBN after amendment addition was first affected by the characteristics of biochar. Biochar, with its extensive surface area and a porous structure, can better coordinate soil water, fertilizer, air, and heat, providing an excellent environment for the growth and reproduction of microorganisms (Clough et al., 2013); also, the surface of biochar has a high density of negative charge that adsorbs substances toxic to microorganisms, which provides a better living environment for the growth of microorganisms and increases microbial biomass (Ok et al., 2015). This also explains why higher contents of M contributed to further increase in MBC and MBN. However, our study showed that MBC and MBN increased up to day 45 and then decreased during the remaining incubation time; this indicated that microorganisms started growing in the presence of easily available organic substrates (de Mora et al., 2005), which were rapidly depleted or stabilized after 45 days. This suggested that biochar did not have the capacity to provide additional substrates for microbial growth, and microorganisms could not achieve continuous increase in this mine soil; in turn, we also confirmed that the addition of M was essential for soil microbial growth (also shown by beta diversity of fungi). In addition, soil microbial growth was not only affected by carbon sources, but also regulated by N fertilizers. In our study, MBC and MBN contents were higher in N2 level, indicating that C/N ratio of 25:1 could satisfy microbial nitrogen demand and contribute to the increase in soil microbial biomass. However, low N and abundant C (C/N ratio of 35:1) may reduce soil microbial biomass.

The level of soil MBC/MBN ratio can reflect the supply capacity of soil N. A small value of MBC/MBN with high bioavailability of nitrogen can improve the utilization rate of soil N (Liang et al., 2006). In our study, the average value of MBC/MBN ratio in all amended treatments was significantly lower than that of the CK treatment, indicating that the combination of amendments could effectively improve the utilization rate of nitrogen. Furthermore, MBC/MBN ratio decreased obviously in N2 levels, in which C+M2+N2 with the lowest ratio. This may be due to the biological activity of N increasing in the combination of biochar and N fertilizer at this level; as a result, more N can be assimilated by the microorganisms, which increases the contents of MBN, resulting in a decrease in the MBC/MBN ratio. It is also possible that N2 level is more conducive to the growth and reproduction of bacterial community, thereby increasing the proportion of bacteria in soil microbial community, and causing a decrease in MBC/MBN ratio due to smaller MBC/MBN ratio in bacterial than fungal community (Tao et al., 2016). Overall, our results confirmed proper C/N ratio of 25:1 (corresponding to biochar + N fertilizer at 1.2 g N/kg soil + microbial agent at 0.8 g/kg) could contribute to the increase in microbial biomass and effectively improve the utilization rate of soil N in short‐term.

### 4.3 Changes in soil bacterial and fungal community composition and structure

The restoration of microbial diversity is a key issue in reclaimed soil systems (Lucas et al., 2014). After a 90‐day incubation, bacterial co‐ordinated alpha and beta diversity were affected by the amendments. The Chao1 estimator of alpha diversity revealed a higher bacterial than fungal species richness in mine soils. Meanwhile, Chao1 estimator of bacterial was significantly higher in N2 level, indicating that proper N addition (corresponding to C/N ratio of 25:1) promoted the restoration of species richness of soil bacterial community. However, Chao1 estimator of fungal was significantly higher in M2 level, indicating that higher M addition promoted the restoration of species richness of soil fungal community. We also observed that bacterial alpha diversity (represented by Simpson indices) in all amended treatments (especially in N2 level) increased significantly compared with CK treatment, while no significant increase was recorded for in soil fungal community, indicating that amended treatments promoted the restoration of species diversity of soil bacterial community, but were not sufficient for increasing that species diversity of soil fungal community.

Beta diversity further indicated that the bacterial community composition formed three separate clusters based on N level, while the fungal community composition was mainly separated by M addition. This indicated that the N level may be a key driving factor affecting bacterial community composition. A possible reason is that soil bacterial community composition may be regulated by C/N ratio by the combined N level and biochar. However, composition of the microbial agent itself may also affect clusters of fungal communities, indicating that fungal community composition was not regulated by biochar and N level but by microbial agent in this study. This finding may attribute to fungi have higher soil nutrient level requirements than we provided by biochar and N fertilizer compared with bacterial community (Niu et al., 2015). The addition of microbial agent affected the composition of soil fungal community, but further studies are needed to confirm this result. Meanwhile, our study also revealed that soil physicochemical properties and microbial biomass together explained a larger proportion of variation in bacterial communities (74.73%) than in fungal communities (35%). This result further confirmed that soil bacteria are highly sensitive to the changes in soil nutrients provided by our amendments (Yao et al., 2014).

In this study, high‐throughput sequencing revealed significant changes in soil bacterial community structure due to the application of amendments at the end of the incubation. The Proteobacteria phyla dominated soil bacterial communities across all soil samples, which was consistent with predominant microbial phyla found in the mining area in a previous study (Kolton et al., 2011; Narendrula‐Kotha & Nkongolo, 2016). This may be related to the extensive degradation properties of Proteobacteria and their ability to inhabit a wide range of habitats (Hanna et al., 2013). At the same time, in our study, the increase over control in the abundances of Proteobacteria phyla with amendments addition may be due to fast growth rates when levels of available substrates are high (Su et al., 2017; Wang et al., 2017; Zhang et al., 2016). Moreover, Figure 5a also showed that the accumulation of soil nutrients provided resources for the survival of Proteobacteria (Fierer et al., 2007). We also found that the relative abundance of Bacteroidetes (*p* < .001) and Firmicutes (*p* < .01) increased significantly in C‐M‐N treatments. Bactericide have fast growth rates and are more likely to grow in eutrophic conditions (Will et al., 2010), which explains the increase in Bacteroidetes in C‐M‐N treatments. Firmicutes have the ability to secrete enzymes which are key to the nitrogen fixation pathway and are directly involved in various other nitrogen metabolism functions (i.e., nitrate reduction, dissimilatory nitrate reduction, and denitrification) (Ren, 2018); thus, Firmicutes are considered to have the potential to promote nitrogen cycling after proper amounts of N fertilizer addition like N1 level in our study. In addition, the relative abundance of Acidobacteria decreased significantly with addition of N fertilizer. The result of RDA also confirmed that the abundance of Acidobacteria was negatively related to TN (Figure 5a). Acidobacteria are generally classified as slow‐growing oligotrophs (Fierer et al., 2007; Wang et al., 2020), and their abundances usually decrease with N fertilizer application (Francioli et al., 2016). Overall, the relative abundance of Bacteroidetes (*p* < .001), Firmicutes (*p* < .01), and Acidobacteria (*p* < .001) changed significantly after proper N addition especially N1 and N2 level (corresponding to C/N ratio of 35:1 and 25:1), indicating that proper C/N ratio (35:1 and 25:1) has a significant effect on the relative abundance of these three bacteria.

The dominant fungal phylum in this study was Ascomycota, corresponding to findings of previous studies in mining soils. Also, the relative abundance of Ascomycota phyla decreased more in C‐M‐N treatments but not significantly, and it also showed a negative correlation with TN (Figure 5b). This result was likely due to the preferred habitat of Ascomycota are particularly important under conditions of low N availability, thereby decline with increased N availability (Beimforde et al., 2014; Yu et al., 2020). Notably, the relative abundance of Chytridiomycota (*p* < .05) decreased significantly at all amended treatments, which may be due to a more sensitive response of Chytridiomycota to changes in soil acidity and nutrient availability. However, the pH was still alkaline in our experiment although it was neutralized after amendments addition. The relative abundance of Basidiomycota, a decomposer of glucose and cellulose, increased after M addition. This may be related to the addition of M which can promote the metabolism of recalcitrant organic carbon by Basidiomycota (Yang et al., 2019).

## 5 CONCLUSIONS

Our results showed relatively higher contents of N and M addition based on biochar in the short‐term can improve soil quality by neutralizing soil pH and increasing soil nutrient contents. N2‐treated soil (corresponding to C/N ratio of 25:1) could contribute to the increase in microbial biomass and the restoration of species richness and diversity (Chao1 estimator, Simpson, and Shannon indices) of soil bacterial community; meanwhile, N2‐ and N1‐treated soil has a significant effect on the relative abundance of Bacteroidetes, Firmicutes, and Acidobacteria, and PCoA further showed that bacterial community composition was regulated by the N level. In addition, RDA analysis indicated that soil bacterial community composition is highly sensitive to the changes in soil nutrients than fungal community composition. These confirmed that adjusting C/N ratio by adding biochar and N fertilizer can affect the composition and diversity of soil bacterial communities. Overall, our study provided a new idea for changing soil microbial community by regulating C/N ratio by amendments to achieve restoration of damaged habitats, which provided a basis for field application to land managers at this coal mine in Qilian Mountains. However, further study is still needed to investigate the response of soil microbial community to long‐term field application of amendments.

## CONFLICT OF INTEREST

The authors declare that they have no conflict of interest.

## AUTHOR CONTRIBUTION

**Junqia Kong:** Conceptualization (equal); Data curation (equal); Formal analysis (equal); Investigation (equal); Methodology (equal); Software (equal); Writing‐original draft (equal); Writing‐review & editing (equal). **Zhibin He:** Formal analysis (equal); Funding acquisition (equal); Project administration (equal); Resources (equal); Supervision (equal). **Longfei Chen:** Conceptualization (equal); Formal analysis (equal); Methodology (equal); Supervision (equal); Validation (equal). **Rong Yang:** Formal analysis (equal); Supervision (equal); Visualization (equal). **Jun Du:** Conceptualization (equal); Formal analysis (equal).

## Supporting information

Appendix S1

## Data Availability

All data are available in the Dryad (https://doi.org/10.5061/dryad.70rxwdbxg).
