# Supplementary material for: Efficiency of biochar, nitrogen addition, and microbial agent amendments in remediation of soil properties and microbial community in Qilian Mountains mine soils
Source: Ecol Evol. 2021 Jun 19;11(14):9318–31. doi: 10.1002/ece3.7715 (PMC8293713; doi:10.1002/ece3.7715)
Supplement: Supplementary file 1 — Appendix S1 [file ECE3-11-9318-s001.docx]

**Appendixes**

**Table S1 Soil physicochemical properties, microbial mass and its ratio in 13 different treatments. Values are means ± standard error, n=15. SOC: soil organic carbon (g kg^-1^), TN: total nitrogen (g kg^-1^), AN: available nitrogen (mg kg^-1^), AP: available phosphorus (mg kg^-1^), AK: available potassium (mg kg^-1^), MBC: microbial biomass carbon (mg kg^-1^), MBN: microbial biomass nitrogen (mg kg^-1^), MBC/MBN: microbial biomass carbon/microbial biomass nitrogen.**

| Treatments | PH | SOC | TN | AN | AP | AK | MBC | MBN | MBC/MBN |
| --- | --- | --- | --- | --- | --- | --- | --- | --- | --- |
| CK | 8.64±0.006abc | 0.71±0.01f | 0.37±0.01e | 17.38±0.30e | 1.9±0.01f | 51.64±0.61g | 49.2±0.77g | 5.15±0.12f | 9.6±0.61a |
| C-N0 | 8.71±0.02ab | 11.92±0.17cd | 1.15±0.11c | 29.86±2.16d | 13.79±0.83e | 166.48±12.36def | 192.64±8.88f | 21.25±1.00e | 9.1±0.74ab |
| C-N1 | 8.55±0.20bcd | 11.68±0.17de | 1.25±0.16bc | 31.9±1.11d | 16.48±0.75de | 150.18±9.49ef | 192.71±9.93f | 21.34±0.66e | 8.98±1.00ab |
| C-N2 | 8.5±0.05cde | 11.61±0.17de | 1.29±0.21bc | 32.75±1.42d | 21.38±0.87ab | 158.4±10.20ef | 220.87±11.86def | 24.74±0.68de | 8.88±1.36ab |
| C-N3 | 8.36±0.08e | 11.39±0.11e | 1.82±0.19c | 34.9±2.16d | 14.25±0.54e | 137.31±5.35f | 200.04±11.46f | 22.5±0.76e | 8.83±1.14ab |
| C-M1-N0 | 8.73±0.03a | 12.26±0.17bc | 1.4±0.17abc | 39.35±5.01cd | 13.98±0.70e | 195.5±15.45bcd | 210.04±10.16ef | 23.22±0.58de | 8.99±1.03ab |
| C-M1-N1 | 8.58±0.06abcd | 12.34±0.22bc | 1.4±0.13abc | 46.99±2.62bc | 17.74±1.15cd | 184.63±13.79cde | 218.98±11.08def | 25.26±0.86d | 8.69±1.56b |
| C-M1-N2 | 8.43±0.09de | 12.34±0.16bc | 1.61±0.15abc | 54.69±6.01ab | 19.4±1.14bc | 264.2±18.39a | 257.5±20.20bc | 29.62±1.08c | 8.46±1.27b |
| C-M1-N3 | 8.36±0.08e | 12.33±0.20bc | 1.83±0.22a | 58.48±5.68a | 14.99±0.91de | 169.71±11.88cdef | 237.07±12.38cde | 28.04±1.26c | 8.65±1.10b |
| C-M2-N0 | 8.71±0.02ab | 12.88±0.21a | 1.47±0.12abc | 47.22±5.23bc | 15.65±0.76de | 224.68±12.77b | 253.42±8.54bcd | 28.98±0.93c | 8.78±0.96ab |
| C-M2-N1 | 8.55±0.06bcd | 12.45±0.19ab | 1.43±0.15abc | 54.98±3.52ab | 15.63±1.25de | 206.48±19.61bc | 236.85±12.63cde | 27.92±1.24c | 8.48±0.79b |
| C-M2-N2 | 8.35±0.08e | 12.54±0.18ab | 1.65±0.17ab | 55.1±4.78ab | 23.48±2.25a | 270.77±17.01a | 302.86±18.22a | 35.88±1.17a | 8.26±1.49b |
| C-M2-N3 | 8.35±0.09e | 12.42±0.23ab | 1.84±0.22a | 60.73±4.06a | 15.22±0.59de | 177.58±13.03cde | 272.81±17.29ab | 32.83±1.11b | 8.42±1.48b |

**Table.S2 Relative abundance of the dominant bacterial and fungal phylum(relative abundance >1%) for all treatments. Values are means ± standard error. Different letters indicate significant differences: * P<0.05, ** P<0.01, *** P<0.001. “-” indicate no significant differences. CK, C, C-N, C-M, C-M-N represent unamended mine soil, biochar, biochar +N fertilizer, biochar + microbial agent, biochar +N fertilizer + microbial agent**

|  | Taxon | CK | C | C-N | C-M | C-M-N | P |
| --- | --- | --- | --- | --- | --- | --- | --- |
| **Bacteria** | Proteobacteria | 73.98±14.44a | 62.69±4.72ab | 67.14±2.32ab | 70.81±5.99a | 59.83±1.77ab | 0.093 |
|  | Actinobacteria | 11.19±8.27a | 14.78±2.61a | 13.62±1.54a | 8.33±2.43a | 10.84±0.66a | 0.296 |
|  | Bacteroidetes | 4.98±1.23b | 6.03±0.67b | 8.88±0.83b | 6.3±0.89b | 12.91±0.98a | ******* |
|  | Gemmatimonadetes | 2.31±1.39b | 4.21±0.94ab | 3.72±0.35ab | 3.26±0.80ab | 5.15±0.54a | 0.091 |
|  | Acidobacteria | 1.37±0.88c | 2.72±0.81a | 0.38±0.10b | 1.85±0.48ac | 0.25±0.05b | ******* |
|  | Chloroflexi | 1.89±1.23ab | 2.32±0.49ab | 1.03±0.09b | 3.05±1.15a | 1.8±0.19ab | 0.102 |
|  | Patescibacteria | 1.23±0.59b | 5.17±2.75a | 2.22±0.51b | 1.7±0.33b | 1.93±0.40b | 0.075 |
|  | Firmicutes | 0.76±0.16b | 0.85±0.31b | 1.85±0.68b | 2.91±0.69b | 4.8±0.63a | 0.003** |
| **Fungal** | Ascomycota | 79.06±1.78a | 85.66±4.46a | 81.53±3.97a | 71.381±6.32a | 69.17±4.13a | 0.196 |
|  | unclassified_Fungi | 6.91±1.82a | 7.61±1.03a | 10.42±4.25a | 10.9±4.81a | 10.22±1.66a | 0.77 |
|  | Basidiomycota | 6.55±1.04b | 4.07±0.34b | 4.77±1.39b | 13.58±6.20a | 9.45±4.52b | 0.798 |
|  | unidentified | 3.15±0.8b | 6.04±2.93ab | 2.48±0.93b | 3.02±0.57b | 10.53±1.70a | **0.005**** |
|  | Chytridiomycota | 2.93±2.93a | 0.2±0.18b | 0.09±0.09b | 0.15±0.11b | 0.07±0.06b | **0.016*** |
